# A Complex Case of Feigned Psychosis or Hidden Truths? A Case Report

**DOI:** 10.1192/j.eurpsy.2025.2046

**Published:** 2025-08-26

**Authors:** M. A. Dimitrov, P. McLaughlin

**Affiliations:** 1 Psychiatry, St James Hospital; 2 Psychiatry, Central Mental Hospital, National Forensic Mental Health Service, Dublin, Ireland

## Abstract

**Introduction:**

Feigning is defined as “to represent falsely; to imitate so as to deceive” (McDermott et al. Int J Law Psychiatry 2013; 36:287-92). Malingering and dissimulation are subtypes of feigning: malingering involves intentionally producing symptoms for incentives (World Health Organization. ICD-11 2022), while dissimulation involves concealing symptoms to appear mentally well (Caruso et al. J Am Acad Psychiatry Law 2003; 31:444-50). The prevalence of feigning illness remains uncertain, and varies with context and incentives. Within the legal context, 17.5% feign incompetence to stand trial and 64.5% to plead not guilty by reason of insanity. Malingering has been reported in up to 56% of general offender samples (McDermott et al. Int J Law Psychiatry 2013; 36:287-92). In the public setting, the malingering prevalence constituted 30% of disability evaluations, 29% of personal injury evaluations, 19% of criminal evaluations and 8% of medical cases (Mittenberg et al. J Clin Exp Neuropsychol). In 2006, malingering resulted in approximately $150 billion in annual expenses for the US insurance industry (Mason et al. Perspect Psychiatr Care 2014; 50: 51-7).

**Objectives:**

To explore the challenges in differentiating psychiatric illness from feigning.

**Methods:**

This case involves analysing the patient’s history, collateral information, and diagnostic interviews to distinguish psychiatric pathology from feigned symptoms.

**Results:**

A 31-year-old male with a history of paranoid schizophrenia, whose recent psychiatric admission was prompted by psychosis and charges of serious assault, property damage, and possession of a weapon. The admission raised suspicions of symptom feigning and patient wariness of the psychiatric stigma. Despite four years of engagement with mental health services (MHS), the patient disclosed shortly after admission that he had been feigning his symptoms to obtain an insanity plea, but now hopes to return to prison seeking a more favourable environment and the certainty of a confirmed guilty sentence. Collateral information from the community MHS and family members suggested underlying psychiatric concerns and manipulative tendencies of the patient, complicating the diagnosis and raising the possibility of dissimulation.

**Conclusions:**

The case highlights the challenges of distinguishing genuine psychiatric illness from deceptive behaviour, emphasizing the importance of thorough history-taking, understanding symptom pathology, using diverse interview techniques, gathering collateral information, and conducting psychological assessments. Clinicians must carefully distinguish feigning from true pathology to provide accurate diagnoses, ensure proper treatment, reduce costs, and safeguard public safety.

**Disclosure of Interest:**

None Declared

